# Cascading Crises: Society in the Age of COVID-19

**DOI:** 10.1177/00027642211003156

**Published:** 2021-11

**Authors:** Laura Robinson, Jeremy Schulz, Christopher Ball, Cara Chiaraluce, Matías Dodel, Jessica Francis, Kuo-Ting Huang, Elisha Johnston, Aneka Khilnani, Oliver Kleinmann, K. Hazel Kwon, Noah McClain, Yee Man Margaret Ng, Heloisa Pait, Massimo Ragnedda, Bianca C. Reisdorf, Maria Laura Ruiu, Cinthia Xavier da Silva, Juliana Maria Trammel, Øyvind N. Wiborg, Apryl A. Williams

**Affiliations:** 1 Santa Clara University, Santa Clara, CA, USA; 2 University of California Berkeley, Berkeley, CA, USA; 3 University of Illinois at Urbana–Champaign, Urbana, IL, USA; 4 Universidad Católica del Uruguay, Montevideo, Uruguay; 5 University of Michigan, Ann Arbor, MI, USA; 6 Ball State University, Muncie, IN, USA; 7 El Camino College, Torrance, CA, USA; 8 The George Washington University, Washington, DC, USA; 9 University of Chicago, Chicago, USA; 10 Arizona State University, Phoenix, AZ, USA; 11 ESMC, New York, NY, USA; 12 São Paulo State University, Julio de Mesquita Filho, São Paulo, Brazil; 13 Northumbria University, Newcastle upon Tyne, UK; 14 University of North Carolina at Charlotte, Charlotte, NC, USA; 15 Department of Education of São Paulo State, Sao Paulo, Brazil; 16 Savannah State University, Savannah, GA, USA; 17 University of Oslo and Oslo Metropolitan University, Oslo, Norway

**Keywords:** COVID-19, pandemic, vulnerability, inequality, resilience

## Abstract

The tsunami of change triggered by the COVID-19 pandemic has transformed society in a series of cascading crises. Unlike disasters that are more temporarily and spatially bounded, the pandemic has continued to expand across time and space for over a year, leaving an unusually broad range of second-order and third-order harms in its wake. Globally, the unusual conditions of the pandemic—unlike other crises—have impacted almost every facet of our lives. The pandemic has deepened existing inequalities and created new vulnerabilities related to social isolation, incarceration, involuntary exclusion from the labor market, diminished economic opportunity, life-and-death risk in the workplace, and a host of emergent digital, emotional, and economic divides. In tandem, many less advantaged individuals and groups have suffered disproportionate hardship related to the pandemic in the form of fear and anxiety, exposure to misinformation, and the effects of the politicization of the crisis. Many of these phenomena will have a long tail that we are only beginning to understand. Nonetheless, the research also offers evidence of resilience on several fronts including nimble organizational response, emergent communication practices, spontaneous solidarity, and the power of hope. While we do not know what the post COVID-19 world will look like, the scholarship here tells us that the virus has not exhausted society’s adaptive potential.

## Cascading Crises

With nearly 100 authors and editors from around the world, our international team has crafted a panorama of the societal impacts of COVID-19 in the first year of the pandemic across thematically aligned issues of the *American Behavioral Scientist*. The research shows a tidal wave of change triggered by the pandemic in ways that would have been unimaginable a year ago. Across the contributions, one of the key themes that emerges—implicitly or explicitly—is the cascading crises wrought by the pandemic. Unlike disasters that are more temporarily and spatially bounded, the pandemic has continued to expand across time and space for over a year, leaving an unusually broad range of second-order and third-order harms in its wake. These issues of *ABS* tackle how the virus has reshaped many facets of society. The first volume, “An Unequal Pandemic: Vulnerability and COVID-19” begins this journey by revealing the unequal effects of the crisis on vulnerable populations. Subsequent volumes explore equally important societal arenas affected by the pandemic including crisis and organizational response, emergent communication practices regarding the virus, the challenges of work and unemployment brought on by COVID-19, and the accelerating impact of digital inequalities. In this special foreword to the collection of international research, we provide highlights from this vast and timely array of scholarly articles.

## Social Isolation and Incarceration

The magnitude of the psychological threat posed by the crisis, as a new form of cultural trauma, is outlined by Seth Abrutyn in his article, “Disintegration in the Age of COVID-19: Collective Trauma, Risk, and the Search for Solidarity.” As Abrutyn points out, the need to protect against contagion prohibits many from seeking community and solidarity. The ongoing enforced isolation is particularly traumatic for those who are socially isolated, such as communities whose sense of well-being stems from strong community ties that have been devastated by COVID-19. The challenges experienced by closely knit communities are also key to the article “The Fine Line: Rural Justice, Public Health and Safety, and the Coronavirus Pandemic” by Jennifer Sherman and Jennifer Schwartz. Their work reveals how resistance to behavioral modification acts against the preservation of public health, particularly with regards to law enforcement and the carceral system for U.S. rural communities prioritizing “freedom and liberty” over public health. Difficult choices to preserve public health and resulting isolation for the incarcerated are also the focus of Bibi Reisdorf’s article “Locked In and Locked Out: How COVID-19 Is Making the Case for Digital Inclusion of Incarcerated Populations.” Reisdorf makes the case that when correctional facilities cut communication, incarcerated people suffer greater isolation and will fare worse upon reentry. As these authors show, a number of unintended negative consequences may be precipitated by public health measures necessary to curb contagion.

## Social Isolation and Age

Social isolation is also one of the cascading secondary effects of the pandemic with respect to older populations as is shown by a series of articles. In “Grey Digital Outcasts and COVID-19,” Simon Rogerson illuminates how older adults have become marked as digital outcasts during the pandemic when so many were forced into physical isolation for the better part of a year. Alexander Seifert and Shelia R. Cotten probe the plight of some of the most isolated older members of society in the article “Digital Distance in Times of Physical Distancing: ICT Infrastructure and Use in Long-Term Care Facilities.” Based on Swiss data, they find that residents suffer from the “double burden of social and digital exclusion,” even in a country with a well-funded safety net. Taken together, these articles illustrate how challenges faced by older adults have been compounded during the pandemic.

## Unemployment and Underemployment

Even those older adults who are still in the labor force experience greater involuntary unemployment and shifts in employment status over the course of the pandemic, as is shown by Phyllis Moen, Joseph H. Pedtke, and Sarah Flood in the article “Derailed by the COVID-19 Economy?”. Yet unemployment, and all of its negative consequences, is not limited to older adults during the crisis. Ofer Sharone describes how job seekers already shut out of the labor market experience even greater stigma while being rebuffed in their job searches during the pandemic in his article “Networking When Unemployed: Why Long-Term Unemployment Will Likely Persist Long After the Covid-19 Pandemic Recedes.” Sharone shows how job seekers’ repetitive “bruising” interactions entail a “direct hit on core bases of identity,” a pattern that is growing more widespread with the prolongation of long-term unemployment caused by the pandemic. Indeed, the “widespread exogenous shock of unemployment” has been experienced far and wide as the pandemic has taken a great toll on the economy, as Heba Gowayed, Ashley Mears, and Nicholas Occhiuto demonstrate in their article “Pause, Pivot, and Shift: Responses to Sudden Job Loss.” They document the extent to which different classes of workers are differentially resilient to pandemic-driven changes in the labor market. On another front, Di Di illuminates how these processes play out among Silicon Valley workers who marshal diverse coping strategies in the face of employment uncertainty in “Surviving is Succeeding: How Tech Workers Handle Job Insecurity during COVID-19.”

## Work

Just as the pandemic intersects with unemployment in complex ways, it has had complex effects on workers and firms across national economies. The unequal effects of public policy regimes in mitigating unemployment forced by lockdowns are also central to the article “The Gendered Politics of Pandemic Relief: Labor and Family Policies in Denmark, Germany, and the United States during COVID-19” by Nino Bariola and Caitlyn Collins. Comparing the social welfare regimes exemplified by these three advanced economies, they identify extensive differences in unemployment relief and child care provisions that can either blunt or sharpen the gendered impact of the pandemic’s economic fallout. Cross-national research is also critical to understanding how context can shape informal work as we see in the article “From Safety Net to Safety Trap: Informality and Telework during the Coronavirus Pandemic in Latin America” by Daniela de los Santos and Inés Fynn. Comparing seven Latin American countries, the article trains attention on the often-neglected informal sector of the economy to show how informal workers are disadvantaged by three factors: ineligibility for pandemic-related government unemployment support, increased exposure to loss of income stemming from shutdowns, and underrepresentation in teleworkable occupations. From a different angle of vision, Steve Viscelli’s “Policy, Worker Power, and the Future of the American Trucker” considers a group of vital essential workers: truckers. Another article probes how the organization of labor and technology are shaping the dynamics of trucking; the research is entitled “Trucking in the Era of COVID-19” by Sperry et al.

## Telework, Gig Work, and Platform Work

In an analogous way, inequality shapes teleworking patterns as is evident in the article “When Lockdowns Force ‘Everyone’ to Work From Home: Inequalities in Telework during COVID-19 in Uruguay” by Matías Dodel and María Julia Acosta. They show how workers’ human capital interacts with employers’ organizational characteristics to create disparities in teleworking patterns. Nonetheless, even in countries with advanced technological infrastructure and governmental promotion of telework, cultural practices may inhibit telework despite its advantages during the pandemic. As Hiroshi Ono argues in his study of Japan entitled “Telework in a Land of Overwork: It’s Not That Simple, or Is It?” one of the main difficulties faced by the Japanese economy is the country’s cultural resistance to telework. Japanese workers and workplaces are singularly unwilling to promote teleworking and remote work arrangements given historical cultural practices and norms in Japanese workplaces. However, even those workers in the most adaptive segments of the economy face new existential threats. In the article “Who Bears the Burden of a Pandemic? COVID-19 and the Transfer of Risk to Digital Platform Workers” by Paula Tubaro and Antonio Casilli, we learn about the consequences of the pandemic for gig workers participating in the relatively new platform economy. The platform economy heightens the economic and health risks posed by COVID-19 for gig workers engaged in public-facing work while allowing teleworkers to shield at home. On a related front, Juliet B. Schor and Mehmet Cansoy explore the impact of the pandemic on platform labor in the “sharing economy” in their article “Commercialization on ‘Sharing Platforms’: the Case of Airbnb Hosting.”

## Risk and Work

The life-and-death risks of public-facing work are also made devastatingly clear in Noah McClain’s article “The Death-Based Model of Organizational Learning: Accident, Pandemic, and Workplace Change in New York Public Transit.” The virus brought an overwhelming loss of life to the ranks of workers who operate New York City’s bus and subway systems, likely attributable to the failure of their employer—the Metropolitan Transportation Authority—to adopt safety reforms until the virus brought a wave of fatalities to its workforce. By linking COVID-19 deaths to analogous pre-COVID-19 fatalities due to the MTA’s long-term poor safety practices, McClain reveals the “death-based model of organizational change” in which organizations systematically fail to take necessary steps to protect their workers until their deaths reach a tipping point. Given the life-and-death implications of public-facing work during the pandemic, many have been forced to make unimaginable choices between health and work as is shown in the article “Emotions during the COVID-19 Crisis: A Health vs. Economy Analysis of Public Responses” by Naga Vemprala, Paras Bhatt, Rohit Valecha, and H. Raghav Rao. Analyzing data from Twitter, they probe emotional responses to health threats versus economic hardship. Shedding light on the difficult decision in which individuals are forced to put either their health or their livelihoods at risk, they offer recommendations on crisis communication relevant to health officials and government agencies.

## Risk and Social Connection

Outside the workplace, social connection also poses new risks according to Xuewen Yan, Tianyao Qu, Nathan Sperber, Jinyuan Lu, Mengzhen Fan, and Benjamin Cornwell in the article “Tied Infections: How Social Connectedness to Other COVID-19 Patients Influences Illness Severity.” Based on public patient-level data from the Chinese city of Shenzhen, their findings show that, for individuals hospitalized with COVID-19, connections to previously ill close ties—whether family or nonfamily—can significantly influence the duration and severity of their sickness. Thus, we can say that the risk of severe disease is at least partially a function of social networks, as holds true in the article “COVID Compatibility and Risk Negotiation in Online Dating during the Pandemic” by Apryl Williams, Gabe Miller, and Guadalupe Marquez-Velarde. The article unpacks how daters engage in risk management using four distinct risk frames: (1) risk tolerance, (2) risk evaluation, (3) risk negotiation, and (4) risk aversion based on gathering information through off-line and online channels before undertaking the risk of physical contact. As Schulz et al. and Schulz show in “Precarity and the Pandemic” and “Risk Governance in Pandemic Society” increased risk has rapidly been normalized across multiple life realms.

## Information Evaluation

Risk assessment relies on access to information. Therefore, information flows are an important form of power and influence that can also perpetuate inequality on many terrains even in highly democratic and economically developed societies. Molly M. King makes this clear in her article “Paying Attention to the Pandemic: Knowledge of COVID-19 by News Sources and Demographics.” The object of this study is to uncover how sociodemographic characteristics—in conjunction with the consumption of specific news sources—affect several types of knowledge relating to the COVID-19 pandemic. King finds that the determinants of knowledge about the government response to COVID-19 differ from the factors influencing knowledge of the medical science underlying the health interventions. Those who rely on nonnational news sources suffer a knowledge gap compared with those who rely on national news outlets, but the gap is more pronounced in terms of local responses as compared with familiarity with public health officials or understanding of medical science. Finally, the relationship between inequality and the use of digital resources to navigate information is also central to the article “COVID-19 Trauma: Problematizing the Pandemic” by authors including Matías Dodel, Noah McClain, and Anabel Quan-Haase. The authors show the ways in which well-being is diminished for those without adequate digital resources and literacies.

## (Mis)Information

Information evaluation skills have become paramount in light of the tsunami of conspiracy theories, misinformation, and rumors generated in response to the pandemic as we see in the article “‘I Heard That COVID-19 Was. . .’: Rumors, Pandemic, and Psychological Distance” by K. Hazel Kwon, Kirstin Pellizzaro, Chun Shao, and Monica Chadha. This article shows how misinformation is assimilated in a crisis where many people are experiencing “an informational malaise.” The analysis finds that psychological distance can explain several kinds of variation in reception patterns to information on social media, including the extent to which the posters considered the conspiracy theories to be true. Misinformation is also the focus of the article “Inoculating an Infodemic: An Ecological Approach to Understanding Engagement with COVID-19 Online Information” by Shandell Houlden, Jaigris Hodson, George Veletsianos, Chris Thompson-Wagner, and Darren Reid. The study adapts what the authors call an ecological understanding of why individuals engage differently with social media content during the COVID-19 crisis. They show that both human and algorithmic forces influence information engagement during the pandemic and make recommendations on mitigating misinformation. These findings are complementary to those in the article “The ‘Parallel Pandemic’ in the Context of China: The Spread of Rumors and Rumor-Corrections during COVID-19 in Chinese Social Media” by Yunya Song, K. Hazel Kwon, Yin Lu, Yining Fan, and Baiqi Li. Their work reveals both how rumors are circulated in Chinese microblogging and how rumors are treated by the social media companies in concert with governmental authorities such that institutional context matters in the correction of rumors.

## Fear

Misinformation may prompt anxiety and fear as the capacity to take advantage of digital resources is indeed a particularly consequential factor influencing individuals’ responses to the pandemic. Yee Man Margaret Ng’s article, “A Cross-National Study of Fear Appeal Messages in YouTube Trending Videos About COVID-19,” applies automated content-analytic methods to analyze transcripts of more than 2000 pandemic-related YouTube videos produced in six countries, including the United States, Brazil, and Taiwan. The study finds a notable imbalance between threat-based messaging and efficacy-based messaging in these videos. The dominance of threat-based messaging was particularly dramatic in the most hard-hit countries. Fear messages are also the topic of the article “Crises Narratives Defining the COVID-19 Pandemic: Expert Uncertainties and Conspiratorial Sensemaking” by Majia Nadesan. Nadesan finds that individuals are confounded in their efforts to understand the virus in light of conflicting information from “experts” that gave rise to collective sensemaking that fostered the growth of conspiratorial narratives. Finally, as David B. Feldman uncovers in “Hope and Fear in the Midst of Coronavirus: What Accounts for COVID-19 Preparedness?” both hope and fear can help predict preparedness and protective behaviors.

## Digital Inequalities

Processes of information evaluation are particularly challenging for the digitally disadvantaged as revealed in the article “The COVID Connection: Pandemic Anxiety, COVID-19 Comprehension, and Digital Confidence” by Laura Robinson, Jeremy Schulz, Øyvind N. Wiborg, and Elisha Johnston. They establish a linkage between digital confidence and increased understanding of the pandemic, as well as a statistically significant relationship in which digital underconfidence predicts somatization of stress or anxiety related to COVID-19 (sweating, trouble breathing, nausea, or a pounding heart). The linkage between stress and digital inequalities also animates the article “The Perceived Impacts of COVID-19 on Users’ Acceptance of Virtual Reality Hardware: A Digital Divide Perspective” by Kuo-Ting Huang, Christopher Ball, and Jessica Francis. They argue that, among technologically oriented individuals, pandemic stress prompts adoption of virtual reality (VR) technology for privileged segments of the population such that COVID-19 is accelerating the adoption of technology and leaving the digitally disadvantaged even further behind. This widening gap is also underscored by the article “COVID-19 Pandemic and eLearning: Digital Divide and Educational Crises in Pakistan’s Universities” by Sadia Jamil and Glenn Muschert. Jamil and Muschert delineate how stay-at-home orders have widened the chasm between the educational experiences of wealthier students in urban areas and those in remote or rural areas who are both economically and technologically disadvantaged, particularly in light of Pakistan’s inadequate technological infrastructure to support remote learning despite eLearning being mandated by the government during the pandemic.

## Markets and Inequalities

In addition to digital inequalities, the pandemic has accentuated the impact of inequality and disadvantage in a variety of market settings. This holds true both for financial and real estate markets as well as labor markets as we saw above. The unequal effects of the pandemic on tenants and renters in the United States is evident in the article “The COVID-19 Pandemic and the Rental Market: Evidence from Craigslist” by John Kuk, Ariela Schachter, Jacob William Faber, and Max Besbris. In this article, the analysis draws on Craigslist data to reveal the interplay of racial/ethnic and sociospatial inequalities as they relate to the trajectories of rents in the 49 largest metropolitan areas in the United States during the pandemic. Moving from real estate to finance, another article turns its attention to the influence of the wealthy and powerful market opportunists who have taken advantage of market opportunities presented by the pandemic-driven economic contraction. As Megan Tobias Neely and Donna Carmichael show in their article “Profiting on Crisis: How Predatory Financial Investors Have Worsened Inequality in the Coronavirus Crisis,” actors in the largely unregulated shadow finance sector (including private equity, venture capital, and hedge fund firms) are enriching themselves during the pandemic-driven recession, often at the expense of low-wage workers and other vulnerable groups in the economy.

## Politicization

Other vulnerable groups have fallen prey to the politicization of the pandemic, as Cristina Flesher Fominaya reveals in the article “Mobilizing during the COVID-19 Pandemic: From Democratic Innovation to the Political Weaponization of Disinformation.” As Fominaya reveals, misinformation presents a real and present danger to democratic institutions where misinformation has grown alongside widespread distrust in “science” as promulgated by institutionalized authorities. As she shows, democratic nations must find new ways to mitigate disinformation without compromising fundamental civil liberties including freedom of speech and the press as they respond to the pandemic, antivaccination campaigns, and other social crises cascading from COVID-19. The impact of the pandemic on the democratic process is also the topic of Juliana Trammel et al.’s article “Politicizing the Pandemic.” Findings map out the divergent informational flows among constituencies on the Twitterverse with regard to the effects of COVID-19 and show the intensification of echo chamber effects in the digital commons. In tandem with Fominaya’s work, Trammel et al’s research shows how the pandemic has intensified ideological polarization.

## Organizational Response

Nimble organization response on the part of activists is the topic of the article “Protest during a Pandemic: How COVID-19 Affected Social Movements in 2020” by Deana A. Rohlinger and David S. Meyer. Chronicling the newly diverse and dispersed activism spurred by an increase in discretionary time, their study uncovers tactical innovation on the part of activists in response to social distancing and public health guidelines including increased use of social media, adding protective gear to public protest, and drive-by campaigns. Also speaking to the success of agile organizational response is the article “U.S. Nonprofit Organizations Respond to the COVID-19 Crisis: Influence of Communication, Crisis Experiences, Crisis Management, and Organizational Characteristics” by Ryan P. Fuller, Ronald E. Rice, and Andrew Pyle, which assesses the responses of nonprofit organizations. Among other findings, they also target effective social media use as a potent medium for nonprofit organizations to affirm their missions, create community, and generate goodwill among various constituencies.

## Zoom: Privacy and Security

Certainly one of the most successful organizations during the pandemic has been Zoom, which has provided a lifeline to the digitally advantaged. However, the skyrocketing use of Zoom and similar interfaces has given rise to new privacy and security concerns as we see in the article “Why Zoom Is Not Doomed Yet: Privacy and Security Crisis Response in the Pandemic” by Wenhong Chen and Yuan Zou. Chen and Zou examine how Zoom was able to quickly respond and adapt the platform to handle privacy and security breaches that occurred in the first months of the pandemic. Tackling Zoom from another angle, Sara Schoonmaker’s article “Navigating Pandemic Crises: Encountering the Digital Commons” raises concerns that must be addressed even after the pandemic is behind us. Schoonmaker focuses on the ability of Zoom-like platforms to facilitate and augment surveillance, which compromises internet users’ privacy across multiple domains. Schoonmaker identifies heightened threats unleashed by the rapacious, commercial profit-seeking model currently dominant in the world of software development. Should these menaces be managed, however, Schoonmaker also ventures in a more hopeful direction by exploring the promise of open-access software platforms and the digital commons as valuable resources during crises such as the COVID-19 pandemic.

## Resilience

Finally, while the pandemic has been the genesis of overwhelming harm, it has also sparked resilience. Among the first examples of hope-inspiring resilience were the spontaneous acts of solidarity in Italy examined by Maria Laura Ruiu and Massimo Ragnedda in the article “Between Online and Offline Solidarity: Lessons Learned from the Coronavirus Outbreak in Italy.” Probing four e-initiatives, they find that social media can generate altruism and community in the face of shared suffering that bridges individuals, families, neighbors, communities, and institutions with a common bond. The resilient use of social media is also central to the article “Piercing the Pandemic Social Bubble: Disability and Social Media Use About COVID-19” by Kerry Dobransky and Eszter Hargittai. Exploring how people with disabilities (PWD) harness social media, they find that PWD were more engaged with information, more active in information sharing, and more likely to support others. Their work offers insights on universal design, “tools long fought for and used by PWD,” and the benefits it offers the general population. Resilient response to the pandemic vis- à-vis eLearning is the theme of the article “Remote Learning: It’s Not All Bad” by Laura Robinson. Examining students’ understanding of their own agency in light of lockdowns mandating remote education, Robinson uncovers the ways in which some students have taken an entrepreneurial approach to remote learning that provides unexpected benefits vis-à-vis sleep, anxiety reduction, and educational agency. Adaptation to changing circumstances is also a common thread in the article “Change in Climate Perception Prompted by the COVID-19 Pandemic” by Gabriele Ruiu et al. They find that an unexpected benefit of the pandemic is emergent awareness of the benefits of sustainable lifestyles. These indications of resilience point to a silver lining in the otherwise bleak panorama of pandemic devastation.

## Society in the Age of COVID-19

Gathered from far and wide, the scholarship assembled in these thematically aligned issues of *ABS* paints a dramatic picture of the cascading crises triggered by COVID-19 that go far beyond the virus itself. As the global case studies show, the pandemic has affected public health, physical and emotional well-being, social relationships, and economic stability for many. While some governments have offered better public health measures and economic safety nets, the unusual conditions of the pandemic-driven social isolation and economic contraction have struck the disadvantaged particularly hard for an extended period of time. Across the articles, we see that the pandemic has deepened existing inequalities and created new vulnerabilities related to loneliness, loss of community, incarceration, involuntary exclusion from the labor market, diminished economic opportunity, and heightened risk of death in the workplace. Many less advantaged individuals and groups have also suffered disproportionate hardship related to the pandemic in the form of fear and anxiety, exposure to misinformation, fallout from the politicization of the crisis, and both digital and economic inequalities. Many of these phenomena have a long tail that we are only beginning to understand. Nonetheless, the research also offers some hope in humanity’s resilience as evidenced by nimble organization response, enhanced forms of communication, and spontaneous solidarity. While we do not know what the post COVID-19 world will look like, the overview of the scholarship here tells us one thing: the virus has not exhausted society’s adaptive potential.

